# Downregulation of KHSRP enhances carboplatin sensitivity in non-small cell lung cancer

**DOI:** 10.1186/s41065-025-00584-4

**Published:** 2025-11-05

**Authors:** Bao Wen, Shuguang Bao, Yanqing Gao, Haoyuan Li, Pengjie Yang, Luri Bao, Chuanhui Teng, Bateer Han

**Affiliations:** 1 Department of Thoracic Tumor Surgery, Peking University Cancer Hospital (Inner Mongolia Campus) & Affiliated Cancer Hospital of Inner Mongolia Medical University, No. 42 Zhao Wu Da Road, Huhhot, 010020 Inner Mongolia Autonomous Region China; 2 Graduate School of Inner Mongolia Medical University, No.5 Xin Hua Road, Huhhot, 010050 Inner Mongolia Autonomous China; 3 Department of Pathology, School of Basic Medical Sciences, Inner Mongolia Medical University, Huhhot, 010107 Inner Mongolia Autonomous Region China

**Keywords:** Non-small cell lung cancer, KHSRP, HMGB1, Carboplatin, Chemotherapy sensitivity

## Abstract

**Background:**

Carboplatin resistance represents a critical therapeutic challenge in non-small cell lung cancer (NSCLC) treatment. Although KHSRP has been implicated in lung cancer progression, its molecular mechanisms and impacts on chemotherapy sensitivity remain elusive. Notably, KHSRP has the capacity to activate the transcription of HMGB1, an oncogene known to influence chemotherapy sensitivity. However, it remains to be determined whether KHSRP affects chemotherapy response in NSCLC via HMGB1.

**Methods:**

KHSRP expression in NSCLC cells was analyzed using qRT-PCR. Cell proliferation, apoptosis, and migration were evaluated using colony formation, flow cytometry and wound healing assays. A luciferase reporter assay was conducted to assess whether KHSRP transcriptionally regulates HMGB1. Additionally, A549 cell xenografts were established in nude mice to investigate the tumor growth-promoting effects of KHSRP in vivo.

**Results:**

KHSRP expression was notably elevated in NSCLC cells. Overexpression of KHSRP remarkably promoted A549 cell proliferation, migration, and epithelial-mesenchymal transition (EMT); while KHSRP knockdown exhibited the opposite effects. Mechanically, KHSRP notably promoted the transcription of HMGB1 and upregulated its expression in A549 cells. Importantly, deficiency of KHSRP remarkably enhanced the suppressive effects of carboplatin on A549 cell proliferation, migration, EMT and HMGB1 expression. Meanwhile, in vivo experiments demonstrated that downregulation of KHSRP potentiated the inhibitory effect of carboplatin on tumor growth in tumor-bearing nude mice.

**Conclusion:**

These findings demonstrate that silencing of KHSRP enhances the drug sensitivity of carboplatin in NSCLC, potentially mediated through the inhibition of HMGB1. Targeting KHSRP may represent a promising therapeutic strategy to improve chemotherapy efficacy in NSCLC.

**Supplementary Information:**

The online version contains supplementary material available at 10.1186/s41065-025-00584-4.

## Introduction

Lung cancer ranks as the most common cancer worldwide and is the primary cause of cancer-related deaths [1]. Based on GLOBOCAN 2022 estimates, approximately 2.5 million new cases and 1.8 million deaths of lung cancer occurred globally in 2022 [1]. Lung cancer is usually divided into non-small cell lung cancer (NSCLC, the main category) and small cell lung cancer (SCLC) [2]. NSCLC can be further divided into subtypes such as adenocarcinoma, squamous cell carcinoma, and large cell carcinoma, with adenocarcinoma being the most common [3]. Patients diagnosed with NSCLC generally have a poor prognosis, with a 5-year overall survival rate of 26.4% [4]. Most patients present with advanced disease or metastasis at the time of diagnosis [5]. The 5-year survival rate for patients with stage IV NSCLC is only 5.8% [4].

At present, the treatment approaches for NSCLC include surgical resection, radiotherapy, chemotherapy, targeted therapy, and immunotherapy [6]. Despite significant progress in targeted therapy and immunotherapy in recent years, platinum-based chemotherapy (cisplatin and carboplatin) continues to serve as the first-line treatment for advanced NSCLC [7, 8]. A randomized study confirmed that the response rate of carboplatin plus paclitaxel chemotherapy for NSCLC could reach 35% [9]. However, the resistance issues that arise during the clinical application of platinum agents seriously affect their efficacy and greatly limit their use. Therefore, developing therapeutic strategies to enhance carboplatin sensitivity may represent a promising approach to overcome clinical resistance.

KH-Type Splicing Regulatory Protein (KHSRP) is a multifunctional RNA-binding protein, which is pivotal in regulating RNA splicing, transport, and editing, as well as mRNA stability and degradation [10]. KHSRP plays an important role in cancer development. Yang et al. found that KHSRP knockdown significantly enhanced the migration ability of glioblastoma multimodal (GBM) cells [11]. However, some studies suggest that KHSRP may exert an opposing role in cancer. Yao et al. found that high KHSRP expression promoted osteosarcoma cells’ proliferation, migration, and invasion [12]. Taniuchi et al. proved that KHSRP downregulation suppressed the invasion and metastasis of pancreatic cancer cells [13]. Tong et al. reported that KHSRP downregulation inhibited cell proliferation and promoted cell apoptosis in vitro, and suppressed tumor growth in vivo of SCLC [14]. Some studies have demonstrated that KHSRP silencing reduced cell proliferation, migration, and invasion in NSCLC, and lung cancer patients with high KHSRP expression had poorer survival [15, 16]. These inconsistencies indicates that the function of KHSRP may be tissue-specific across different tumors. However, the molecular mechanism of KHSRP in NSCLC progression and its impact on chemotherapy sensitivity in NSCLC have not been elucidated.

In this study, we analyzed the role of KHSRP in proliferation, migration and apoptosis of NSCLC cells, and evaluated its regulatory effect on the sensitivity of NSCLC cells to carboplatin. Our data indicated that the expression of KHSRP was upregulated in NSCLC cells. Downregulation of KHSRP suppressed the proliferation and migration and triggered apoptosis of NSCLC cells. Importantly, KHSRP knockdown enhanced the carboplatin sensitivity of NSCLC cells. Our research may provide a theoretical basis for overcoming carboplatin resistance of NSCLC in clinic.

## Materials and methods

### Cell culture

Human normal bronchial epithelial cell line (16HBE, BNCC338044) and NSCLC cell lines, A549 (BNCC337696), H1299 (BNCC100268), and H1975 (BNCC340345), were obtained from BNCC. 16HBE cells were cultured in complete KM medium containing 1% antibiotics (penicillin/streptomycin) and 10% fetal bovine serum (FBS). A549 cells were cultured in complete F-12 K medium containing 1% antibiotics (penicillin/streptomycin) and 10% FBS. H1975 and H1299 were cultured in complete RPMI-1640 medium containing 1% antibiotics (penicillin/streptomycin) and 10% FBS. All cells were incubated at 37 °C in a 5% CO_2_ incubator.

### Lentivirus production and infection

The sequences of KHSRP shRNAs were cloned into HBLV-U6-MCS-CMV-ZsGreen PGK-PURO vector (Hanbio) to generate KHSRP knockdown lentivirus vectors (sh-KHSRP-1 and sh-KHSRP-2). The full-length KHSRP sequence was amplified by PCR and cloned into pHBLV-CMV-MCS-3flag-EF1-mCherry-T2A puro vector (Hanbio) to generate KHSRP overexpression lentivirus vector (lenti-KHSRP). Next, the above vector plasmids, psPAX2 (Hanbio), and pMD2G (Hanbio) plasmids were co-transfected into HEK-293T cells using Lipofiter™ transfection reagent (Hanbio). Virus supernatants were harvested at 48 and 72 h after transfection, and then used to infect A549 cells for 48 h. After infection, the stable cells were screened with puromycin.

### Cell treatment

A549 cells were transfected with NC, sh-KHSRP-1, sh-KHSRP-2, or lenti-KHSRP for 24 h. Additionally, A549 cells were treated with different concentrations of carboplatin (0, 10, 20, 40, 60 and 80 µg/mL) for 24, 48 and 72 h. Meanwhile, to evaluate the role of KHSRP in carboplatin sensitivity, A549 cells were transfected with either sh-KHSRP-2 or lenti-KHSRP. After incubation for 6 h at 37 °C, the medium was replaced with fresh complete F-12 K medium and the cells were then exposed to 20 µg/mL carboplatin for 72 h.

### Quantitative real-time polymerase chain reaction (qRT-PCR)

Redzol reagent (SBS Genetech), SureScriptTM First-Strand cDNA Synthesis Kit (GeneCopeia) and 2×SYBR Green qPCR Master Mix (Servicebio) were used for RNA extraction, reverse transcription, and qPCR amplification in accordance with the manufacturer’s instructions, respectively. The mRNA expression of KHSRP was calculated using 2^−ΔΔCT^ method with GAPDH as the internal control. The primes were as follows: GAPDH: forward, 5’-AATCCCATCACCATCTTCC-3’, reverse, 5’-GAGTCCTTCCACGATACCAA-3’; KHSRP: forward, 5’-CCGAGGTGGCGAGAATGT-3’, KHSRP reverse, 5’-GCTGCTTTGCTTGGGTCA-3’.

### Western blotting

Protein was isolated using RIPA lysis buffer (Biorigin) containing PMSF (Biorigin). Protein concentration was measured using BCA protein assay kit (Utibody). Protein was separated with 5–10% sodium dodecyl sulfate-polyacrylamide gel electrophoresis, which was transferred to polyvinylidene fluoride membrane (Millipore). Then, the membranes were blocked in 5% defatted milk and incubated overnight at 4 °C with primary antibodies against KHSRP (CST, 1:1000), HMGB1 (Thermofisher, 1:800), N-cadherin (Thermofisher, 1:1000), E-cadherin (Thermofisher, 1:1000), vimentin (Thermofisher, 1:5000), Ki67 (Thermofisher, 1:100), GAPDH (Abcam, 1:2500), and β-actin (Abcam, 1:1000). The membranes were incubated with the Goat Anti-Rabbit IgG H&L (HRP) secondary antibody (Abcam, 1:8000) at room temperature after being washed. Lastly, the protein blots was visualized by hemiScope6100 chemiluminescence imaging system (Clinx). For data quantification and normalization, signal intensity of the target protein was normalized to that of loading control protein (GAPDH/β-actin).

### Cell counting Kit-8 (CCK-8) assay

Cell viability was measured by CCK-8 (Beyotime). A549 cells (4000 cells per well) were plated on 96-well plates overnight at 37 °C, and subjected to different treatments. Next, 10 µL of CCK8 solution was added and then incubated for 2 h at 37 °C. The absorbance was measured at 450 nm.

### Colony formation assay

After transfection or pharmaceutical treatment, A549 cells (600 cells per well) were seeded and cultured in 12-well plates for 7 days. The colonies were counted after staining with 0.1% crystal violet for 3 min. Images were taken with a digital camera. The colonies exhibiting intense crystal violet staining were quantified after staining.

### Apoptosis detection by flow cytometric analysis

Cell apoptosis was evaluated using Annexin V-FITC/PI apoptosis kit (LiankeBio) following the manufacturer’s protocol. A549 cells were stained with 5 µL Annexin V-FITC and 10 µL propidium iodide (PI), and then incubated at room temperature in the dark for 20 min. Cell apoptosis rate was detected using a FACSCalibur™ flow cytometer (BD Biosciences).

### Wound healing assay

After transfection or pharmaceutical treatment, A549 cells (5*10^4^ cells per well) were seeded on 6-well plates and cultured to near 80% to 90% confluence. The monolayers were scratched with a sterile pipette tip, and the floating cells were removed with PBS. Next, cells were then cultured in serum free medium. The images were taken at 0, 24, and 48 h. The spacing (pixel spacing) between scratches in each field of view was measured using Image J software at each time point.

###  Transwell invasion assay

Cell invasion was evaluated with a 24-well transwell chamber (Corning). The upper chamber membrane was pre-coated with 60 µL of Matrigel per well (Matrigel: medium = 1:8). A total of 10^5^ cells were suspended in 200 µL of serum-free medium. Subsequently, the cell suspensions were added to the upper chamber, while the lower chamber was filled with 500 µL of medium supplemented with 10% FBS. After 24 h of incubation, the cells that had invaded to the underside of the membrane were stained with 0.1% crystal violet and photographed for quantification.

### Luciferase reporter assay

The wild-type (wt) and mutant (mut) promoter sequences of HMGB1 were inserted into pmirGLO luciferase plasmid (Hongke) to generate HMGB1-wt and HMGB1-mut reporter vectors. Then, HMGB1 reporter vectors and lenti-KHSRP or sh-KHSRP-2 plasmids were co-transfected into A549 cells using Lipofectamine 2000 (Invitrogen). After 48 h of transfection, Dual-Luciferase Reporter Assay System (Beyotime) was used to detect the luciferase activity.

### Animal experiments

The animal experiments were conducted in accordance with the guidelines by the Tianjin Medical Experimental Animal Care. Approval for the animal protocols was granted by the Institutional Animal Care and Use Committee at Yi Shengyuan Gene Technology (Tianjin) Co., Ltd. (number YSY-DWLL-2024726). Male BALB/c nude mice (6–8 weeks old) from SiPeiFu (Beijing) were raised under controlled conditions of temperature (23 ± 2 °C), humidity (50 ± 5%), and cyclic light (light/dark 10/14 hours) [17]. All mice were divided into six groups randomly: negative control (NC), NC + carboplatin, sh-KHSRP-2, sh-KHSRP-2 + carboplatin, lenti-KHSRP, and lenti-KHSRP + carboplatin. Each group contained five mice. Mice were subcutaneously injected with 1 × 10^7^ NC-, sh-KHSRP-2-, or lenti-KHSRP-transfected A549 cells. Mice in the carboplatin treatment group were administered intraperitoneally with 15 mg/kg carboplatin (twice or thrice a week). Tumor growth was monitored once a week for 4 consecutive weeks. The tumor size was calculated as: tumor volume = tumor length x tumor width^2^/2. After 4 weeks, mice were sacrificed by cervical dislocation under anesthesia (1% isoflurane, inhalation), and tumors were harvested and weighted.

### Hematoxylin and Eosin (H&E) staining

Tumor tissues excised from mice were fixed overnight in 4% paraformaldehyde. Then, these tissues were embedded in paraffin and sliced into 4 μm sections. After dewaxing, the sections were subjected to hematoxylin and eosin staining. The sections were scanned using the 3DHISTECH panoramic scanner (PANNORAMIC). Meanwhile, the image capture was performed at 20x objective magnification.

### TUNEL assay

The TUNEL assay was performed following the kit protocol (Servicebio). The sections of tumor tissues were deparaffinized using xylene, and incubated with 100 µL of proteinase K (20 µg/mL) for 20 min at 37 °C. These sections were incubated with 50 µL of Equilibration Buffer for 30 min, and then incubated with 56 µL of mixed buffer (Recombinant TDT enzyme: CF488-Dutp Labeling Mix: Equilibration Buffer = 1:5:50) for 1 h at 37 °C. DAPI staining solution was added dropwise onto the sections in the dark. Finally, images were captured using a panoramic scanner (3DHISTECH, Budapest, Hungary).

### Statistical analysis

All data were reported as mean ± standard deviations (SD). Each experiment was performed in three biologically independent replicates. Data were analyzed using Prism version 8 (GraphPad). One-way analysis of variance with Tukey’s test were used to analyze the significance of differences between three or more groups. *P* < 0.05 indicated statistical difference.

## Results

### KHSRP expression is upregulated in NSCLC cells

**Fig. 1:**
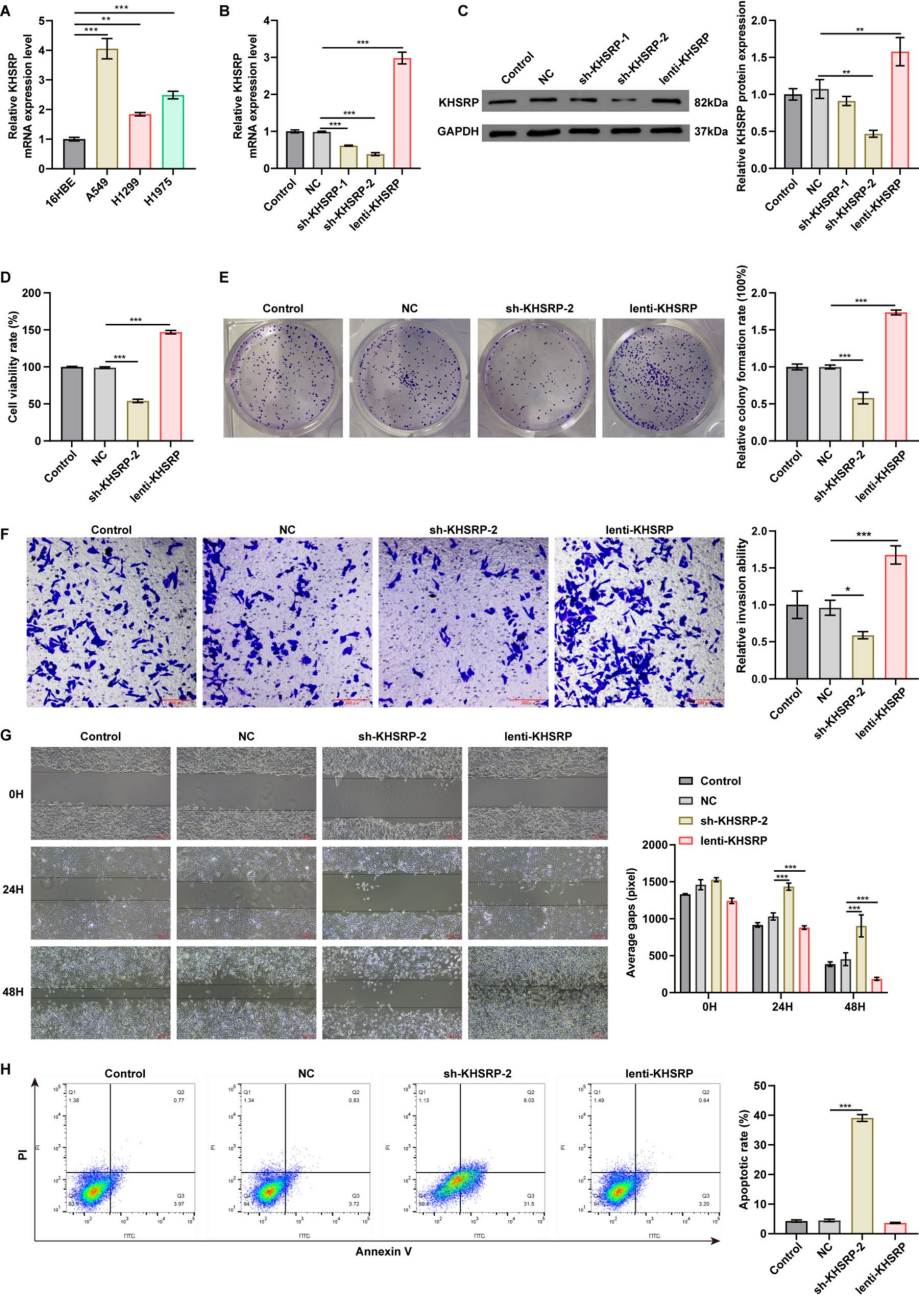
KHSRP expression was elevated in NSCLC cells, and modulation of KHSRP expression level impacted the viability, proliferation, migration and apoptosis of NSCLC cells. (**A**) qRT-PCR analysis showed elevated KHSRP mRNA levels in NSCLC cell lines. (**B-C**) qRT-PCR and Western blot analyses of KHSRP levels in A549 cells infected with lentiviruses carrying lenti-con (NC), sh-KHSRP-1, sh-KHSRP-2, or lenti-KHSRP for 24 h. Sh-KHSRP-1 and sh-KHSRP-2 reduced KHSRP levels, whereas lenti-KHSRP elevated KHSRP levels. (C) For Western blot results, the relative protein expression levels of KHSRP were normalized to GAPDH; the results are presented as mean ± SD. The blot images are presented as images from different membranes. (**D**) A549 cells were infected with NC, sh-KHSRP-2, or lenti-KHSRP for 24 h. CCK-8 assay showed that sh-KHSRP-2 suppressed cell viability, whereas lenti-KHSRP promoted cell viability. (**E**) Colony formation assay revealed that sh-KHSRP-2 suppressed cell proliferation, whereas lenti-KHSRP promoted cell proliferation. (**F**,** G**) Wound healing and transwell invaison assays indicated that sh-KHSRP-2 suppressed cell migration and invasion, whereas lenti-KHSRP promoted cell migration and invasion. (**H**) Flow cytometry analysis indicated that sh-KHSRP-2 induced cell apoptosis. *P<0.05, **P < 0.01, ***P < 0.001

Firstly, we determined the expression of KHSRP in NSCLC cell lines. The results showed that compared with 16HBE cells, the expression of KHSRP was significantly increased in A549, H1299, and H1975 cells (Fig. 1A). As A549 cells exhibited the highest KHSRP mRNA levels, they were selected for subsequent functional studies.

Compared to the NC group, KHSRP expression was significantly downregulated in A549 cells transfected with sh-KHSRP-1 or sh-KHSRP-2, whereas it was increased in cells transfected with lenti-KHSRP (Fig. 1B and C). Since sh-KHSRP-2 exhibited a stronger silencing effect, it was chosen for subsequent experiments (Fig. 1B and C).

### KHSRP enhances proliferative, migratory, and invasive capacities of NSCLC cells

- To evaluate the functional roles of KHSRP in NSCLC, we conducted CCK-8, colony formation, flow cytometry, wound healing and transwell invasion assays. Remarkably, overexpression of KHSRP enhanced A549 cell viability, proliferation, migration and invasion (Fig. 1D-G). Conversely, knockdown of KHSRP notably reduced A549 cell viability, proliferation, migration and invasion, but triggered cell apoptosis (Fig. 1D-H). To sum up, KHSRP exhibits the pro-tumorigenic effects in NSCLC cells.

### KHSRP increased the transcription of HMGB1 and upregulated HMGB1 gene expression

**Fig. 2:**
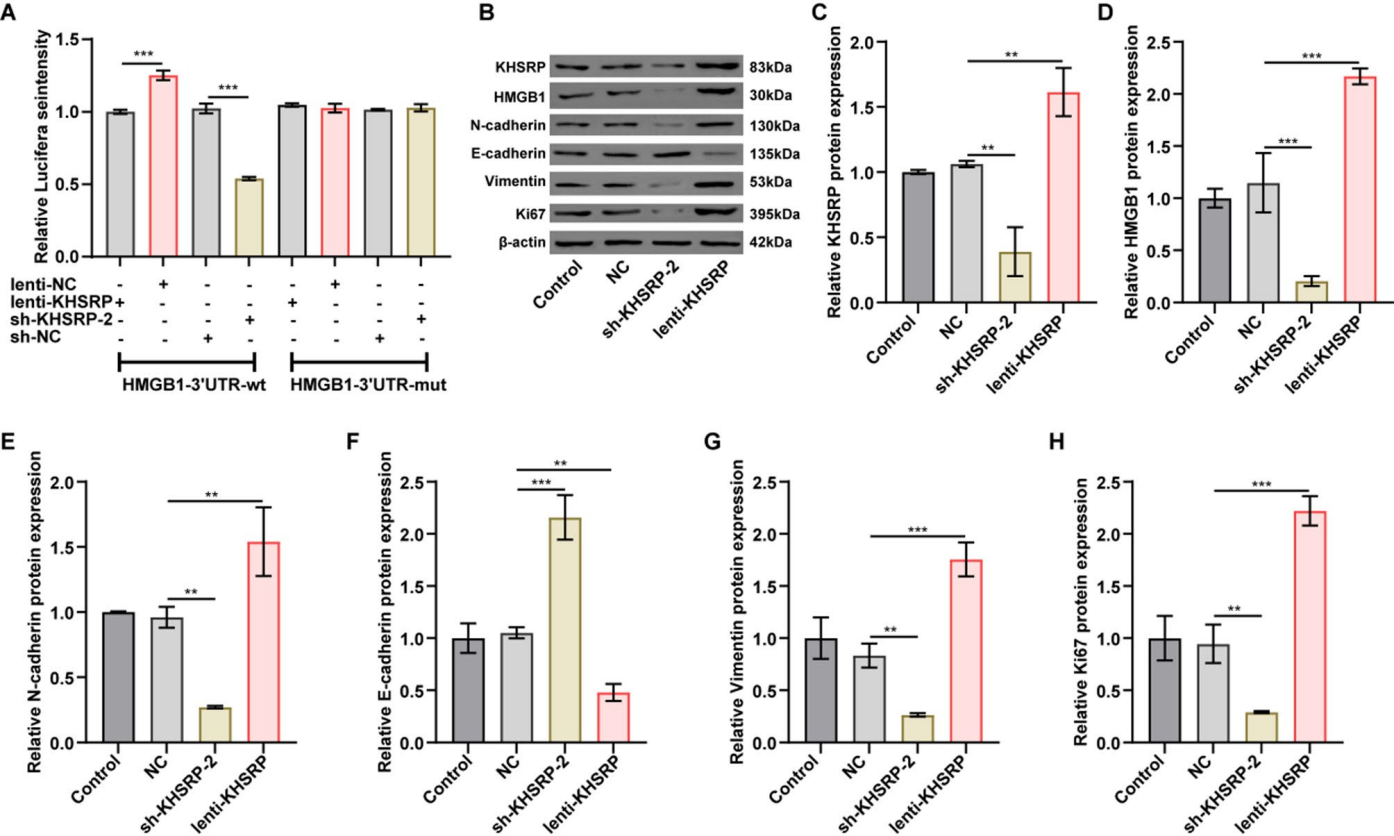
KHSRP promoted HMGB1 transcription and EMT process in NSCLC cells.(**A**) The luciferase activity was detected in A549 cells transfected with HMGB1-WT/MUT plasmids and lenti-KHSRP-OE or sh-KHSRP-2. (**B-H**) A549 cells were infected with NC, sh-KHSRP-2, or lenti-KHSRP for 24 h. Western blot analysis indicated that overexpression or knockdown of KHSRP altered the protein expression levels of KHSRP, HMGB1, N-cadherin, E-cadherin, vimentin, and Ki67 in A549 cells. The relative expression levels of these proteins were normalized to β-actin; the results are presented as mean ± SD. The blot images are presented as images from different membranes. **P < 0.01, ***P < 0.001

The results of luciferase reporter assay showed that knockdown of KHSRP significantly reduced the luciferase activity of the reporter gene driven by the HMGB1-WT promoter; conversely, overexpression of KHSRP significantly increased the HMGB1 promoter luciferase reporter gene activity (Fig. 2A). These findings indicate that KHSRP enhances the transcriptional activity of HMGB1 in A549 cells, which is consistent with previous reports in a prior study showing that KHSRP can activate the transcription of HMGB1 [18].

In addition, Western blot results showed that KHSRP knockdown significantly reduced KHSRP and HMGB1 expression levels in A549 cells, while KHSRP overexpression displayed the opposite effects (Fig. 2B-D). Collectively, KHSRP can promote the transcription and expression of HMGB1.

### KHSRP promoted the epithelial-mesenchymal transition (EMT) in NSCLC cells

Next, we investigated the potential influence of KHSRP on the expression levels of EMT markers (epithelial markers: E-cadherin; mesenchymal markers: N-cadherin and vimentin) and proliferation-associated protein Ki67 in A549 cells. As indicated in Fig. 2B, E-H, downregulation of KHSRP resulted in a notable increase in E-cadherin levels and a significant decrease in the expression of N-cadherin, vimentin and Ki67 in A549 cells when compared to the NC group; in contrast, KHSRP overexpression had opposing effects. Collectively, these findings suggest that KHSRP can modulate the expression of EMT and proliferation markers in A549 cells.

### KHSRP knockdown enhanced carboplatin sensitivity of NSCLC cells

**Fig. 3:**
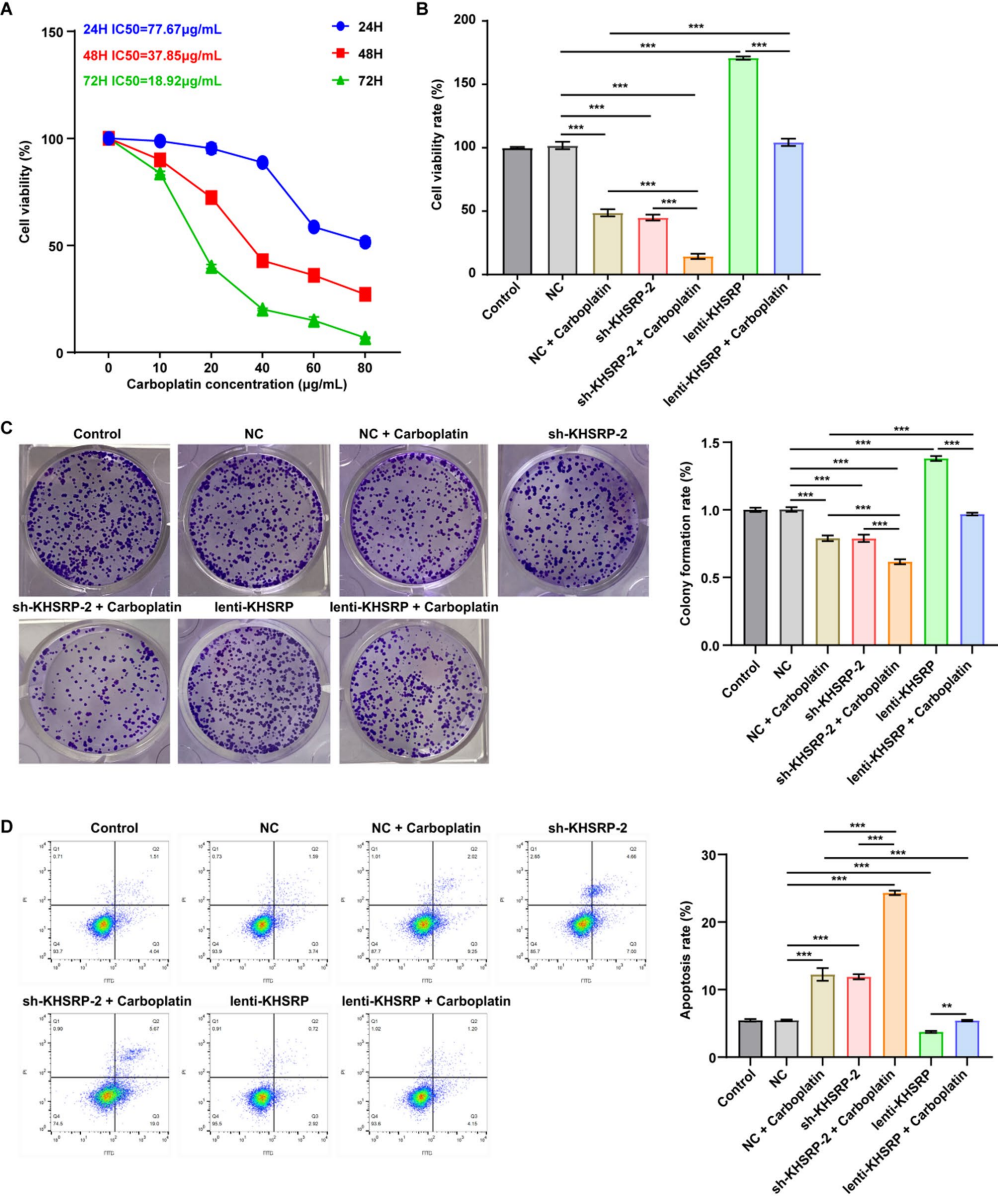
Combination of carboplatin and KHSRP knockdown inhibited the proliferation and induced apoptosis of NSCLC cells.(**A**) A549 cells were treated with different concentrations of carboplatin for 24, 48 and 72 h. CCK-8 assay showed that carboplatin inhibited cell viability in a time-dependent and dose-dependent manner. (**B**) A549 cells infected with NC, sh-KHSRP-2, or lenti-KHSRP were exposed to 20 µg/mL carboplatin for 72 h. CCK-8 assay indicated that knockdown of KHSRP potentiated the cytotoxicity of carboplatin in A549 cells, and its overexpression diminished it. (**C**) Colony formation assay showed that knockdown of KHSRP potentiated the anti-proliferative effect of carboplatin in A549 cells, and its overexpression mitigated it. (**D**) Flow cytometry analysis revealed that knockdown of KHSRP potentiated the pro-apoptotic effect of carboplatin in A549 cells, and its overexpression mitigated it. ***P < 0.001

We next to explore whether KHSRP can affect carboplatin sensitivity in NSCLC. This line of inquiry was motivated by the fact that carboplatin-based chemotherapy is the first-line treatment option for advanced NSCLC patients [7, 8], yet the development of chemoresistance is a primary cause of treatment failure. Elevating drug sensitivity may represent a promising therapeutic approach in oncology. As shown in Fig. 3A, carboplatin inhibited the cell viability of A549 cells in a time-dependent and dose-dependent manner. The half-maximal inhibitory concentrations (IC₅₀) of carboplatin were determined to be 77.67 µg/mL at 24 h, 37.85 µg/mL at 48 h, and 18.92 µg/mL after 72 h of treatment (Fig. 3A). Based on these results, a concentration of 20 µg/mL was selected for subsequent experiments, as it reduced cell viability to approximately 50% following 72 h of exposure (Fig. 3A).

**Fig. 4:**
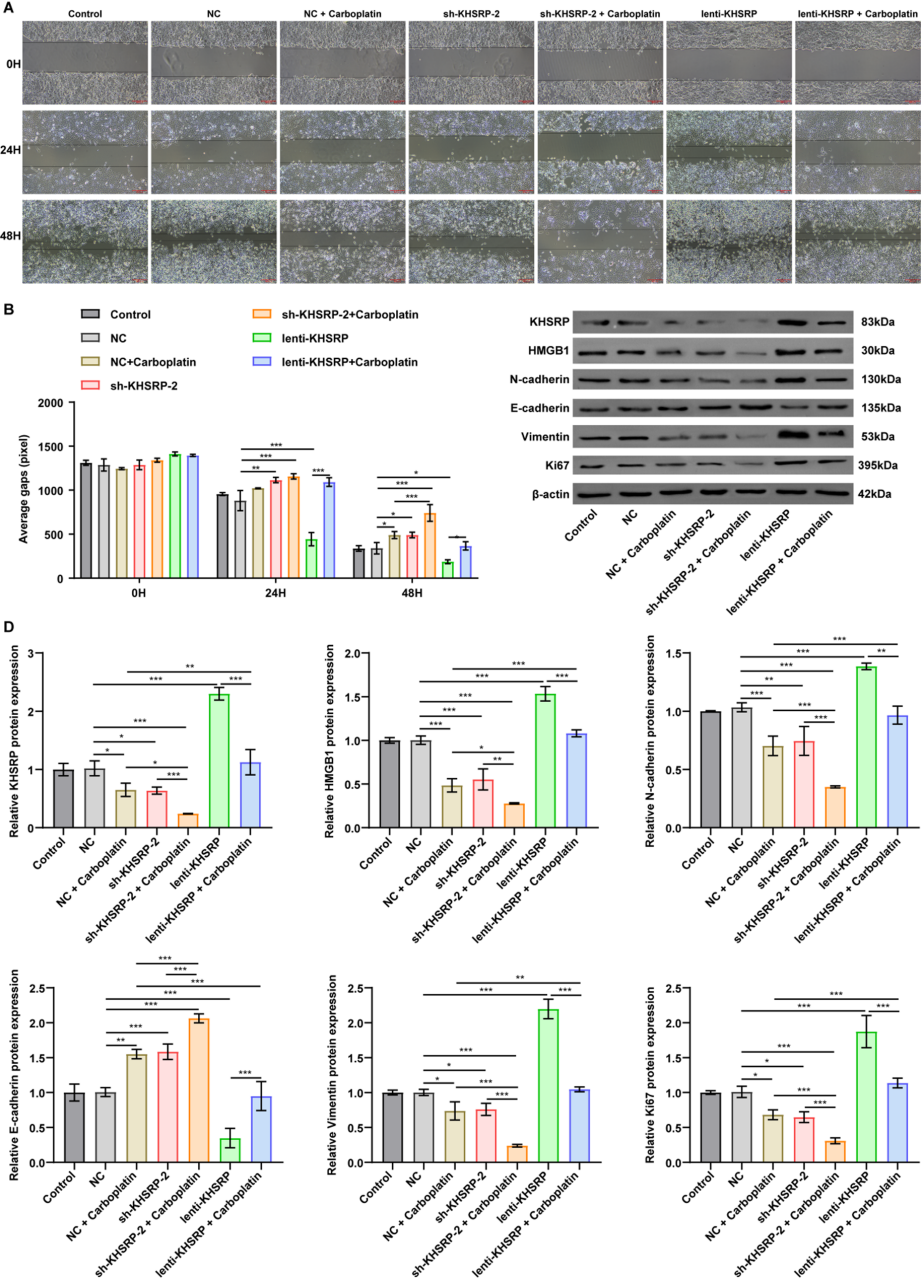
Combination of carboplatin and KHSRP knockdown suppressed migration and EMT process in NSCLC cells. A549 cells infected with NC, sh-KHSRP-2, or lenti-KHSRP were exposed to 20 µg/mL carboplatin for 72 h. (**A-B**) Wound healing assay showed that knockdown of KHSRP potentiated the anti-migratory effect of carboplatin in A549 cells, and its overexpression mitigated it. (**C-D**) Western blot analysis indicated that carboplatin treatment, KHSRP overexpression, or KHSRP knockdown altered the expression levels of KHSRP, HMGB1, N-cadherin, E-cadherin, vimentin, and Ki67 levels in A549 cells. The relative protein expression levels of these proteins were normalized to β-actin; the results are presented as mean ± SD. The blot images are presented as images from different membranes. *P < 0.05, **P < 0.01, ***P < 0.001

As shown in Figs. 3B-D and 4A-B, carboplatin treatment significantly reduced A549 cell viability, proliferation and migration, but induced cell apoptosis, compared to NC group. Notably, silencing KHSRP further amplified these effects, leading to a more pronounced suppression of viability, proliferation and migration, as well as an upregulation of apoptosis compared to NC + carboplatin treatment group (Figs. 3B-D and 4A-B). Conversely, overexpression of KHSRP markedly diminished the anti-proliferative, anti-migratory and pro-apoptotic effects of carboplatin on A549 cells (Figs. 3B-D and 4A-B). Furthermore, compared to NC group, both carboplatin and KHSRP knockdown notably reduced KHSRP and HMGB1 expression levels, as well as decreased N-cadherin, vimentin and ki67 and increased E-cadherin expression levels in A549 cells; conversely, KHSRP overexpression elicited opposing effects (Fig. 4C-D). Notably, compared to NC + carboplatin group, combination of sh-KHSRP-2 and carboplatin further diminished the expression of KHSRP, HMGB1, N-cadherin, vimentin, and Ki67, and increased E-cadherin levels in A549 cells (Fig. 4C-D). To sum up, knockdown of KHSRP has the potential to enhance the sensitivity of A549 cells to carboplatin.

### KHSRP knockdown enhanced carboplatin sensitivity of NSCLC cells in vivo

**Fig. 5:**
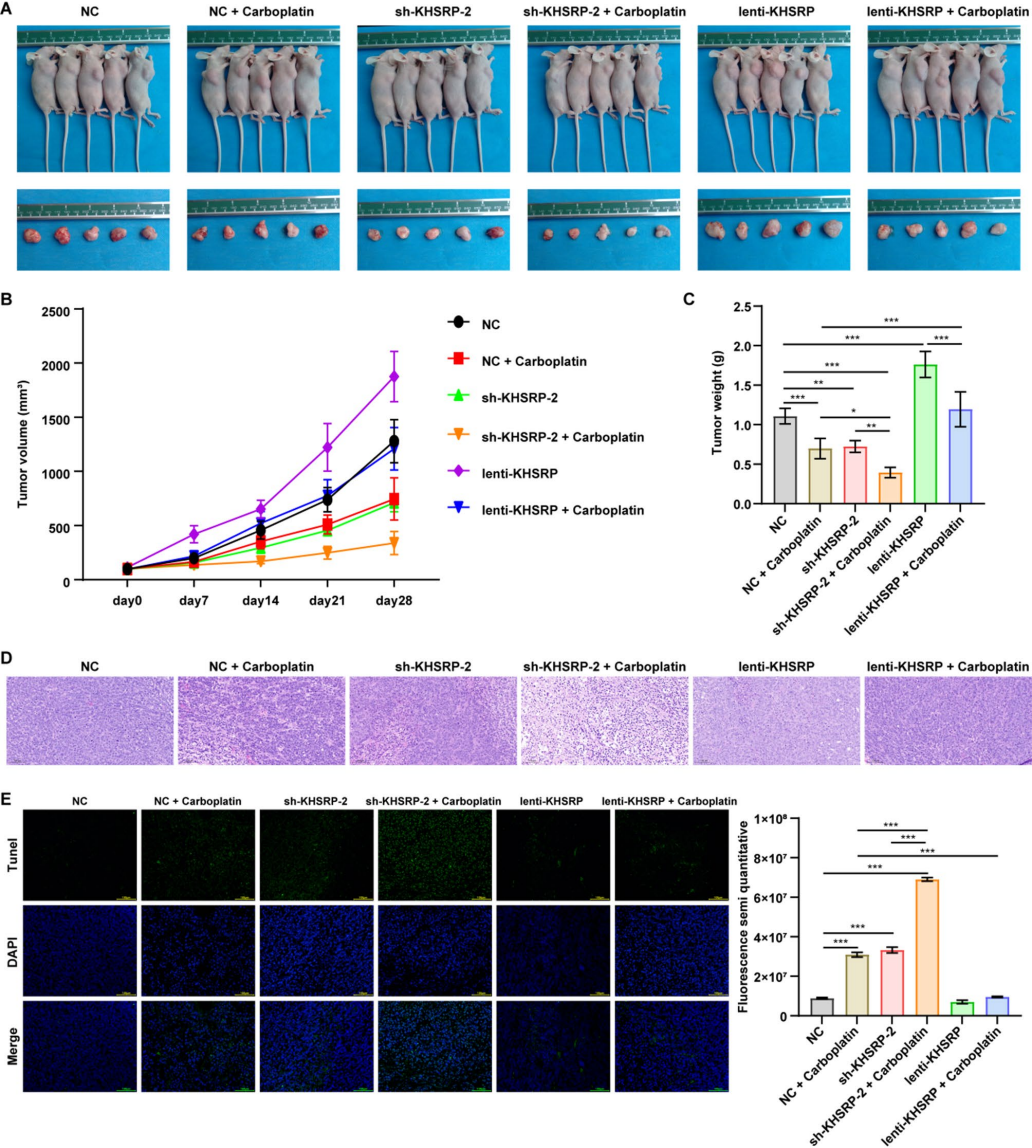
KHSRP knockdown enhanced carboplatin sensitivity of NSCLC cells in vivo. (**A**) The representative photographs of tumor-bearing nude mice and resected tumors at 28 days. (**B**) The tumor volumes in different treatment groups were measured at specified time points. Combination of carboplatin and sh-KHSRP-2 showed the strongest inhibitory effect on tumor volume. (**C**) The tumor weights in different treatment groups were measured. Combination of carboplatin and sh-KHSRP-2 demonstrated the strongest inhibitory effect on tumor weight. (**D**) H&E staining analysis showed that combination of carboplatin and sh-KHSRP-2 remarkably caused the pathological changes in tumor tissues. (**E**) TUNEL assay indicated that combination of carboplatin and sh-KHSRP-2 strongly induced tumor cell apoptosis. *P < 0.05, **P < 0.01, ***P < 0.001

To assess the combined antitumor efficacy of carboplatin and KHSRP downregulation in vivo, we established a A549 cell-derived xenograft mouse model. The results showed that both KHSRP knockdown and carboplatin treatment significantly suppressed tumor growth, as evidenced by reduced tumor volume and weight (Fig. 5A-C). As expected, the combination of KHSRP knockdown and carboplatin treatment further reduced tumor volume and weight compared to either treatment alone (Fig. 5A-C). Conversely, KHSRP overexpression mitigated the tumor-suppressive effects of carboplatin when compared to the NC + carboplatin group (Fig. 5A-C). H&E staining of tumor sections showed that combined KHSRP knockdown and carboplatin treatment markedly increased tumor necrotic areas compared to either sh-KHSRP-2 or NC + carboplatin treatment groups (Fig. 5D). Additionally, TUNEL assay results suggested that the combination of KHSRP knockdown and carboplatin treatment significantly increased cell apoptosis in tumor tissues relative to single treatments (Fig. 5E). In contrast, KHSRP overexpression reduced carboplatin-induced cell apoptosis in tumor tissues when compared to the NC + carboplatin group (Fig. 5E).

**Fig. 6:**
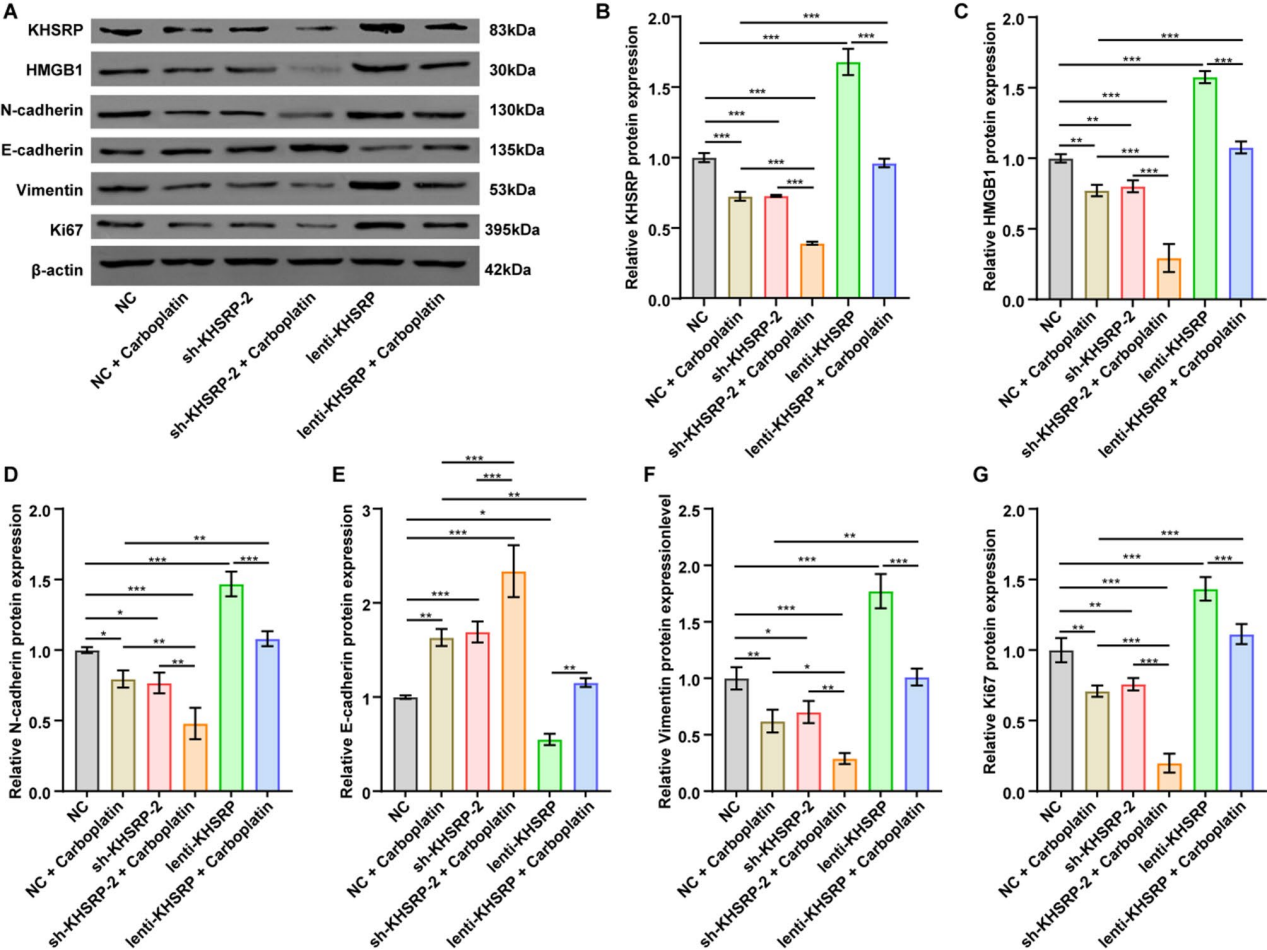
KHSRP knockdown enhanced carboplatin sensitivity of NSCLC cells in vivo through inhibiting HMGB1 expression and EMT process. (**A-G**) Western blot analysis indicated that carboplatin treatment, KHSRP overexpression, or KHSRP knockdown altered the expression levels of KHSRP, HMGB1, N-cadherin, E-cadherin, vimentin, and Ki67 levels in tumor tissues of tumor-bearing mice. The relative protein expression levels of these proteins were normalized to β-actin; the results are presented as mean ± SD. The blot images are presented as images from different membranes. *P < 0.05, **P < 0.01, ***P < 0.001

Furthermore, compared to single treatments, the combination of carboplatin and KHSRP knockdown further reduced KHSRP, HMGB1, N-cadherin, vimentin, and Ki67 levels, and increased E-cadherin levels in mouse tumor tissues (Fig. 6A-G). Notably, when combined with NC + carboplatin group, KHSRP overexpression substantially reversed carboplatin-induced alterations in these proteins (Fig. 6A-G). These in vivo results further suggest a potential association between KHSRP expression and carboplatin sensitivity in NSCLC.

## Discussion

In this study, we determined that the KHSRP expression was significantly higher in NSCLC cells than in normal bronchial epithelial cells. KHSRP overexpression promoted the proliferation and migration of NSCLC cells in vitro, as well as the tumor growth in vivo. Notably, we discovered for the first time that KHSRP overexpression also could reduce the sensitivity of A549 cells to carboplatin both in vitro and in vivo. These findings indicate that KHSRP plays a critical role in affecting carboplatin sensitivity in NSCLC.

Mechanistically, the results of the luciferase reporter assay demonstrated that KHSRP activated HMGB1 transcription in A549 cells, consistent with a previous study by Xu et al. [18]. Meanwhile, KHSRP overexpression significantly increased HMGB1 expression in A549 cells; whereas, KHSRP silencing exhibited the opposite effects. HMGB1 is a non-histone chromosomal protein that functions differently at different subcellular or extracellular locations [19, 20]. Under normal conditions, HMGB1 in the nucleus is involved in DNA organization and transcriptional regulation. When cells are stimulated, HMGB1 is passively released or actively secreted into the extracellular space. Extracellular HMGB1 acts as a damage-associated molecular pattern (DAMP) regulating inflammatory immune responses. Increasing evidence indicates that HMGB1 is significantly associated with the occurrence and progression of NSCLC tumors [21–23]. Wu et al. demonstrated that HMGB1 overexpression has the potential to promote lung cancer cell migration and invasion [24]. Wang et al. indicated that forced expression of HMGB1 was able to facilitate proliferation and metastasis in lung cancer through upregulation of Wnt/β-Catenin signaling [25]. This evidence indicates that HMGB1 operates as an oncogene in lung cancer. Therefore, we propose that KHSRP may play an oncogenic role in NSCLC by increasing the transcription of HMGB1. Furthermore, HMGB1 has also been found to influence chemosensitivity or resistance to chemotherapy in NSCLC. Shen et al. observed that HMGB1 could enhance the chemoresistance in small cell lung cancer through enhancing PARP1-related nucleophagy [26]. Ma et al. reported that HMGB1 overexpression was capable of diminishing the sensitivity of NSCLC cells to cisplatin [27]. Our results indicated that carboplatin significantly reduced the expression levels of KHSRP and HMGB1 in A549 cells. However, the overexpression of KHSRP notably reversed the alterations in these proteins induced by carboplatin. Based on these findings, we speculate that KHSRP influences carboplatin sensitivity in NSCLC by modulating the transcription and expression levels of HMGB1.

EMT is a phenomenon in which epithelial cells transform into mesenchymal phenotype under the influence of multiple factors [28, 29]. In NSCLC, the occurrence of EMT promotes cell invasion and metastasis. Restoring E-cadherin expression was able to suppress the invasion and migration potential of NSCLC cells [30]. Meanwhile, inhibition of N-cadherin expression could block the proliferation and invasion of NSCLC cells [31]. Furthermore, EMT is also a cause of drug resistance in NSCLC. Studies have shown that docetaxel-resistant NSCLC cells exhibited typical features of mesenchymal phenotype compared to parental cells, including spindle-shaped cell morphology, loss of cell adhesion, and increased formation of pseudopodia [32, 33]. Moreover, the E-cadherin expression in docetaxel-resistant cells decreased, while the expression of fibronectin, N-cadherin, and vimentin increased [32, 33]. These findings imply that EMT is closely associated with tumor progression and drug resistance in NSCLC. Our study indicated that KHSRP knockdown could inhibit the EMT process in A549 cells, as evidenced by the reduced expression of N-cadherin and vimentin, alongside an increase in E-cadherin. Moreover, KHSRP knockdown was able to further enhance the inhibitory effects of carboplatin on EMT in A549 cells, suggesting that downregulation of KHSRP could enhance carboplatin sensitivity in NSCLC through the inhibition of EMT. Furthermore, HMGB1 has been shown to promote EMT in various human cancers, such as osteosarcoma and colorectal cancer [34, 35]. Thus, we suspected that deficiency of KHSRP may suppress tumor progression and enhance the sensitivity of NSCLC cells to carboplatin by inhibiting EMT through downregulation of HMGB1 expression; whereas the precise underlying mechanisms require further investigation in future studies.

## Conclusion

In summary, we have determined that KHSRP was overexpressed in NSCLC cells, and is essential for driving proliferation, migration, and EMT. Meanwhile, we have demonstrated for the first time that KHSRP silencing could enhance carboplatin sensitivity of NSCLC cells through inhibition of HMGB1 transcription. These findings suggest that KHSRP may serve as a promising therapeutic target to improve chemotherapy efficacy in NSCLC patients, offering novel insights into the molecular mechanisms underlying tumor progression and chemosensitivity.

## Supplementary Information

Supplementary Material 1.

Supplementary Material 2.

## Data Availability

The original contributions presented in the study are included in the article.

## References

[1] Bray F, Laversanne M, Sung H, Ferlay J, Siegel RL, Soerjomataram I, et al. Global cancer statistics 2022: GLOBOCAN estimates of incidence and mortality worldwide for 36 cancers in 185 countries. CA Cancer J Clin. 2024;74(3):229–63. 10.3322/caac.21834. 38572751 10.3322/caac.21834

[2] Herbst RS, Heymach JV, Lippman SM. Lung cancer. N Engl J Med. 2008;359(13):1367–80. 10.1056/NEJMra0802714. 18815398 10.1056/NEJMra0802714 PMC10662965

[3] Singh T, Fatehi Hassanabad M, Fatehi Hassanabad A. Non-small cell lung cancer: emerging molecular targeted and immunotherapeutic agents. Biochim Biophys Acta Rev Cancer. 2021;1876(2):188636. 10.1016/j.bbcan.2021.188636. 34655692 10.1016/j.bbcan.2021.188636

[4] Ganti AK, Klein AB, Cotarla I, Seal B, Chou E. Update of Incidence, Prevalence, Survival, and initial treatment in patients with Non-Small cell lung cancer in the US. JAMA Oncol. 2021;7(12):1824–32. 10.1001/jamaoncol.2021.4932. 34673888 10.1001/jamaoncol.2021.4932 PMC8532041

[5] Crino L, Weder W, van Meerbeeck J, Felip E, Group EGW. Early stage and locally advanced (non-metastatic) non-small-cell lung cancer: ESMO clinical practice guidelines for diagnosis, treatment and follow-up. Ann Oncol. 2010;21(Suppl 5):v103–15. 10.1093/annonc/mdq207. 20555058 10.1093/annonc/mdq207

[6] Alduais Y, Zhang H, Fan F, Chen J, Chen B. Non-small cell lung cancer (NSCLC): A review of risk factors, diagnosis, and treatment. Medicine. 2023;102(8):e32899. 10.1097/MD.0000000000032899. 36827002 10.1097/MD.0000000000032899 PMC11309591

[7] Planchard D, Popat S, Kerr K, Novello S, Smit EF, Faivre-Finn C, et al. Metastatic non-small cell lung cancer: ESMO clinical practice guidelines for diagnosis, treatment and follow-up. Ann Oncol. 2018;29(Suppl 4):iv192–237. 10.1093/annonc/mdy275. 30285222 10.1093/annonc/mdy275

[8] Hanna N, Johnson D, Temin S, Baker S Jr., Brahmer J, Ellis PM, et al. Systemic therapy for stage IV Non-Small-Cell lung cancer: American society of clinical oncology clinical practice guideline update. J Clin Oncol. 2017;35(30):3484–515. 10.1200/JCO.2017.74.6065. 28806116 10.1200/JCO.2017.74.6065

[9] Sandler A, Gray R, Perry MC, Brahmer J, Schiller JH, Dowlati A, et al. Paclitaxel-carboplatin alone or with bevacizumab for non-small-cell lung cancer. N Engl J Med. 2006;355(24):2542–50. 10.1056/NEJMoa061884. 17167137 10.1056/NEJMoa061884

[10] Gherzi R, Lee KY, Briata P, Wegmuller D, Moroni C, Karin M, et al. A KH domain RNA binding protein, KSRP, promotes ARE-directed mRNA turnover by recruiting the degradation machinery. Mol Cell. 2004;14(5):571–83. 10.1016/j.molcel.2004.05.002. 15175153 10.1016/j.molcel.2004.05.002

[11] Yang J, Fan J, Li Y, Li F, Chen P, Fan Y, et al. Genome-wide RNAi screening identifies genes inhibiting the migration of glioblastoma cells. PLoS ONE. 2013;8(4):e61915. 10.1371/journal.pone.0061915. 23593504 10.1371/journal.pone.0061915 PMC3625150

[12] Yao XY, Liu JF, Luo Y, Xu XZ, Bu J. LncRNA HOTTIP facilitates cell proliferation, invasion, and migration in osteosarcoma by interaction with PTBP1 to promote KHSRP level. Cell Cycle (Georgetown Tex). 2021;20(3):283–97. 10.1080/15384101.2020.1870820. 33475442 10.1080/15384101.2020.1870820 PMC7889103

[13] Taniuchi K, Ogasawara M. KHSRP-bound small nucleolar RNAs associate with promotion of cell invasiveness and metastasis of pancreatic cancer. Oncotarget. 2020;11(2):131–47. 10.18632/oncotarget.27413. 32010427 10.18632/oncotarget.27413 PMC6968780

[14] Tong L, Luo Y, Wei T, Guo L, Wang H, Zhu W, et al. KH-type splicing regulatory protein (KHSRP) contributes to tumorigenesis by promoting miR-26a maturation in small cell lung cancer. Mol Cell Biochem. 2016;422(1–2):61–74. 10.1007/s11010-016-2806-y. 27644194 10.1007/s11010-016-2806-y

[15] Yan M, Sun L, Li J, Yu H, Lin H, Yu T, et al. RNA-binding protein KHSRP promotes tumor growth and metastasis in non-small cell lung cancer. J Exp Clin Cancer Res. 2019;38(1):478. 10.1186/s13046-019-1479-2. 31775888 10.1186/s13046-019-1479-2 PMC6882349

[16] Bikkavilli RK, Zerayesus SA, Van Scoyk M, Wilson L, Wu PY, Baskaran A, et al. K-homology splicing regulatory protein (KSRP) promotes post-transcriptional destabilization of Spry4 transcripts in non-small cell lung cancer. J Biol Chem. 2017;292(18):7423–34. 10.1074/jbc.M116.757906. 28275056 10.1074/jbc.M116.757906 PMC5418043

[17] Wang Q, Sun Z, Du L, Xu C, Wang Y, Yang B, et al. Melatonin sensitizes human colorectal cancer cells to gamma-ray ionizing radiation in vitro and in vivo. Int J Mol Sci. 2018;19(12):3974. 10.3390/ijms19123974 10.3390/ijms19123974 PMC6320774 30544713

[18] Xu J, Wang D, Ma H, Zhai X, Huo Y, Ren Y, et al. KHSRP combines transcriptional and posttranscriptional mechanisms to regulate monocytic differentiation. Blood Sci. 2022;4(3):103–15. 10.1097/BS9.0000000000000122. 36518592 10.1097/BS9.0000000000000122 PMC9742092

[19] Tang D, Kang R, Zeh HJ, Lotze MT. The multifunctional protein HMGB1: 50 years of discovery. Nat Rev Immunol. 2023;23(12):824–41. 10.1038/s41577-023-00894-6. 37322174 10.1038/s41577-023-00894-6

[20] Chen R, Kang R, Tang D. The mechanism of HMGB1 secretion and release. Exp Mol Med. 2022;54(2):91–102. 10.1038/s12276-022-00736-w. 35217834 10.1038/s12276-022-00736-w PMC8894452

[21] Xia Q, Xu J, Chen H, Gao Y, Gong F, Hu L, et al. Association between an elevated level of HMGB1 and non-small-cell lung cancer: a meta-analysis and literature review. Onco Targets Ther. 2016;9:3917–23. 10.2147/OTT.S104409. 27418836 10.2147/OTT.S104409 PMC4935082

[22] Jakubowska K, Naumnik W, Niklinska W, Chyczewska E. Clinical significance of HMGB-1 and TGF-beta level in serum and BALF of advanced Non-Small cell lung cancer. Adv Exp Med Biol. 2015;852:49–58. 10.1007/5584_2015_115. 25753556 10.1007/5584_2015_115

[23] Wang JL, Wu DW, Cheng ZZ, Han WZ, Xu SW, Sun NN. Expression of high mobility group box - B1 (HMGB-1) and matrix metalloproteinase-9 (MMP-9) in non-small cell lung cancer (NSCLC). Asian Pac J Cancer Prevention: APJCP. 2014;15(12):4865–9. 10.7314/apjcp.2014.15.12.4865. 10.7314/apjcp.2014.15.12.4865 24998555

[24] Wu X, Wang W, Chen Y, Liu X, Wang J, Qin X, et al. Glycyrrhizin suppresses the growth of human NSCLC cell line HCC827 by downregulating HMGB1 level. Biomed Res Int. 2018;2018:6916797. 10.1155/2018/6916797. 29568761 10.1155/2018/6916797 PMC5820661

[25] Wang XH, Zhang SY, Shi M, Xu XP. HMGB1 promotes the proliferation and metastasis of lung cancer by activating the Wnt/beta-Catenin pathway. Technol Cancer Res Treat. 2020;19:1533033820948054. 10.1177/1533033820948054. 32815451 10.1177/1533033820948054 PMC7444109

[26] Shen W, Lyu Q, Yi R, Sun Y, Zhang W, Wei T, et al. HMGB1 promotes chemoresistance in small cell lung cancer by inducing PARP1-related nucleophagy. J Adv Res. 2024;66:165–80. 10.1016/j.jare.2023.12.020. 38159843 10.1016/j.jare.2023.12.020 PMC11674788

[27] Ma Y, Kang S, Wu X, Han B, Jin Z, Guo Z. Up-regulated HMGB1 in the pleural effusion of non-small cell lung cancer (NSCLC) patients reduces the chemosensitivity of NSCLC cells. Tumori. 2018;104(5):338–43. 10.5301/tj.5000656. 28885675 10.5301/tj.5000656

[28] Yang J, Antin P, Berx G, Blanpain C, Brabletz T, Bronner M, et al. Guidelines and definitions for research on epithelial-mesenchymal transition. Nat Rev Mol Cell Biol. 2020;21(6):341–52. 10.1038/s41580-020-0237-9. 32300252 10.1038/s41580-020-0237-9 PMC7250738

[29] Lamouille S, Xu J, Derynck R. Molecular mechanisms of epithelial-mesenchymal transition. Nat Rev Mol Cell Biol. 2014;15(3):178–96. 10.1038/nrm3758. 24556840 10.1038/nrm3758 PMC4240281

[30] Mateen S, Raina K, Agarwal C, Chan D, Agarwal R. Silibinin synergizes with histone deacetylase and DNA methyltransferase inhibitors in upregulating E-cadherin expression together with Inhibition of migration and invasion of human non-small cell lung cancer cells. J Pharmacol Exp Ther. 2013;345(2):206–14. 10.1124/jpet.113.203471. 23461975 10.1124/jpet.113.203471 PMC3629797

[31] Zhang X, Liu G, Kang Y, Dong Z, Qian Q, Ma X. N-cadherin expression is associated with acquisition of EMT phenotype and with enhanced invasion in erlotinib-resistant lung cancer cell lines. PLoS ONE. 2013;8(3):e57692. 10.1371/journal.pone.0057692. 23520479 10.1371/journal.pone.0057692 PMC3592915

[32] Shen W, Pang H, Liu J, Zhou J, Zhang F, Liu L, et al. Epithelial-mesenchymal transition contributes to docetaxel resistance in human non-small cell lung cancer. Oncol Res. 2014;22(1):47–55. 10.3727/096504014X14098532393473. 25700358 10.3727/096504014X14098532393473 PMC7592784

[33] Cui SY, Huang JY, Chen YT, Song HZ, Feng B, Huang GC, et al. Let-7c governs the acquisition of chemo- or radioresistance and epithelial-to-mesenchymal transition phenotypes in docetaxel-resistant lung adenocarcinoma. Mol Cancer Research: MCR. 2013;11(7):699–713. 10.1158/1541-7786.MCR-13-0019-T. 23562878 10.1158/1541-7786.MCR-13-0019-T

[34] Hou C, Lu M, Lei Z, Dai S, Chen W, Du S et al. HMGB1 positive feedback loop between cancer cells and Tumor-Associated macrophages promotes osteosarcoma migration and Invasion. Laboratory investigation; a journal of technical methods and pathology. 2023;103(5):100054. 10.1016/j.labinv.2022.100054 10.1016/j.labinv.2022.100054 36801636

[35] Gao X, Zhou S, Qin Z, Li D, Zhu Y, Ma D. Upregulation of HMGB1 in tumor-associated macrophages induced by tumor cell-derived lactate further promotes colorectal cancer progression. J Translational Med. 2023;21(1):53. 10.1186/s12967-023-03918-w. 10.1186/s12967-023-03918-w PMC9883966 36709284

