# Supplementary material for: Downregulation of KHSRP enhances carboplatin sensitivity in non-small cell lung cancer
Source: Hereditas. 2025 Nov 5;162:224. doi: 10.1186/s41065-025-00584-4 (PMC12587549; doi:10.1186/s41065-025-00584-4)
Supplement: Supplementary file 1 — Supplementary Material 1. [file 41065_2025_584_MOESM1_ESM.docx]

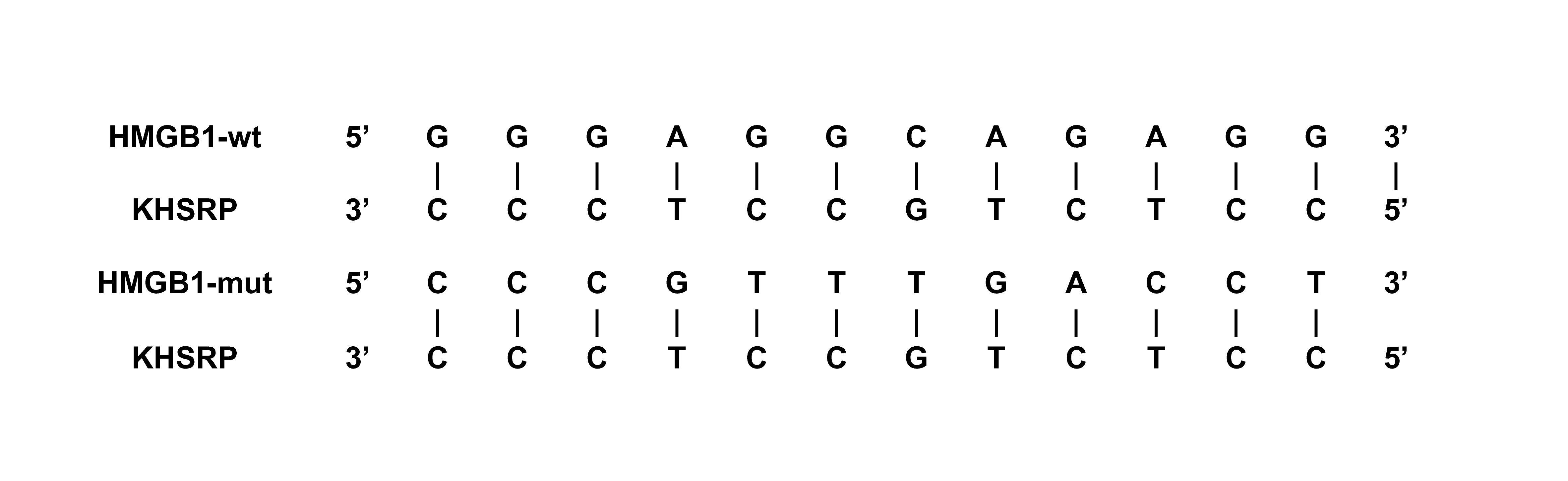


**Supplementary figure 1** Wild‐type sequence (HMGB1-wt) and mutated sequence (HMGB1-mut) for KHSRP binding site.
