# Supplementary material for: Downregulation of KHSRP enhances carboplatin sensitivity in non-small cell lung cancer
Source: Hereditas. 2025 Nov 5;162:224. doi: 10.1186/s41065-025-00584-4 (PMC12587549; doi:10.1186/s41065-025-00584-4)

Figure 1C

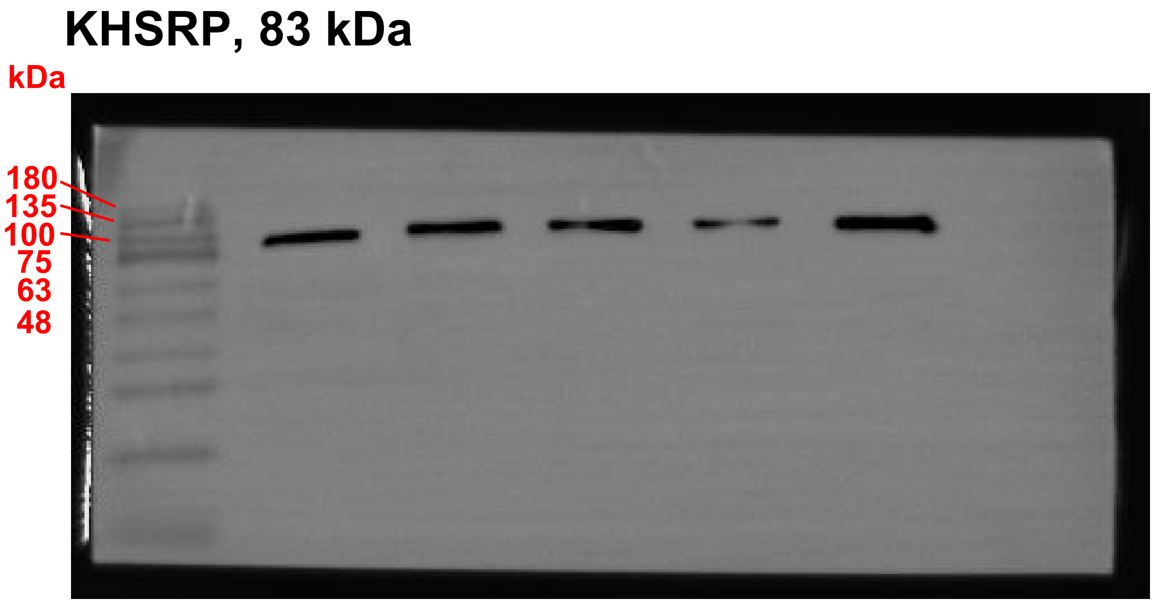
KHSRP, 83 kDa
kDa
180
135
100
75
63
48

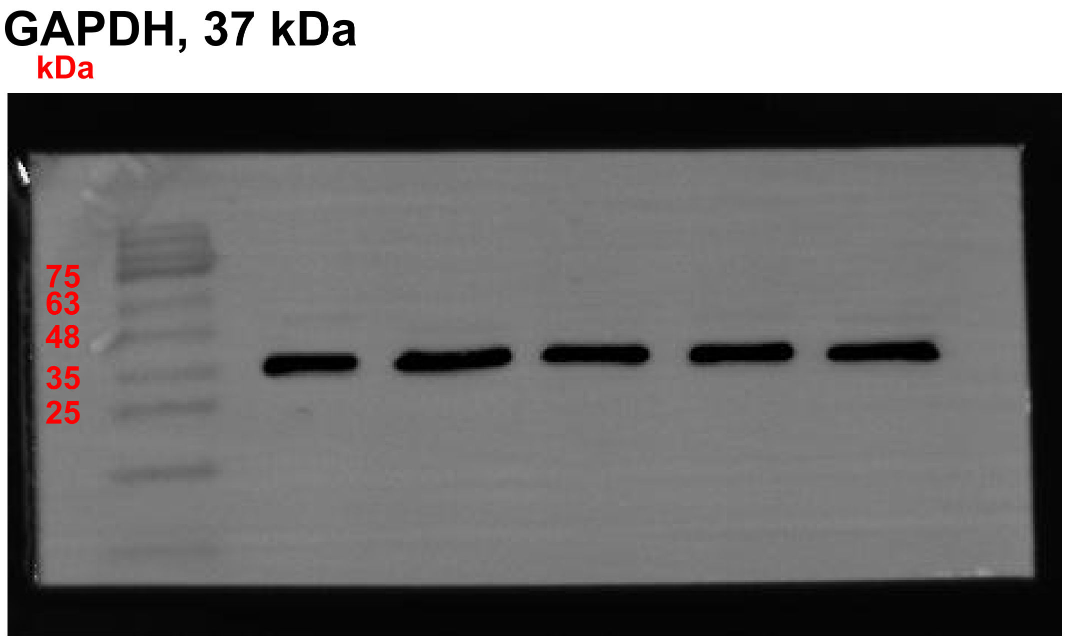
GAPDH, 37 kDa
kDa
75
63
48
35
25

Figure 2B

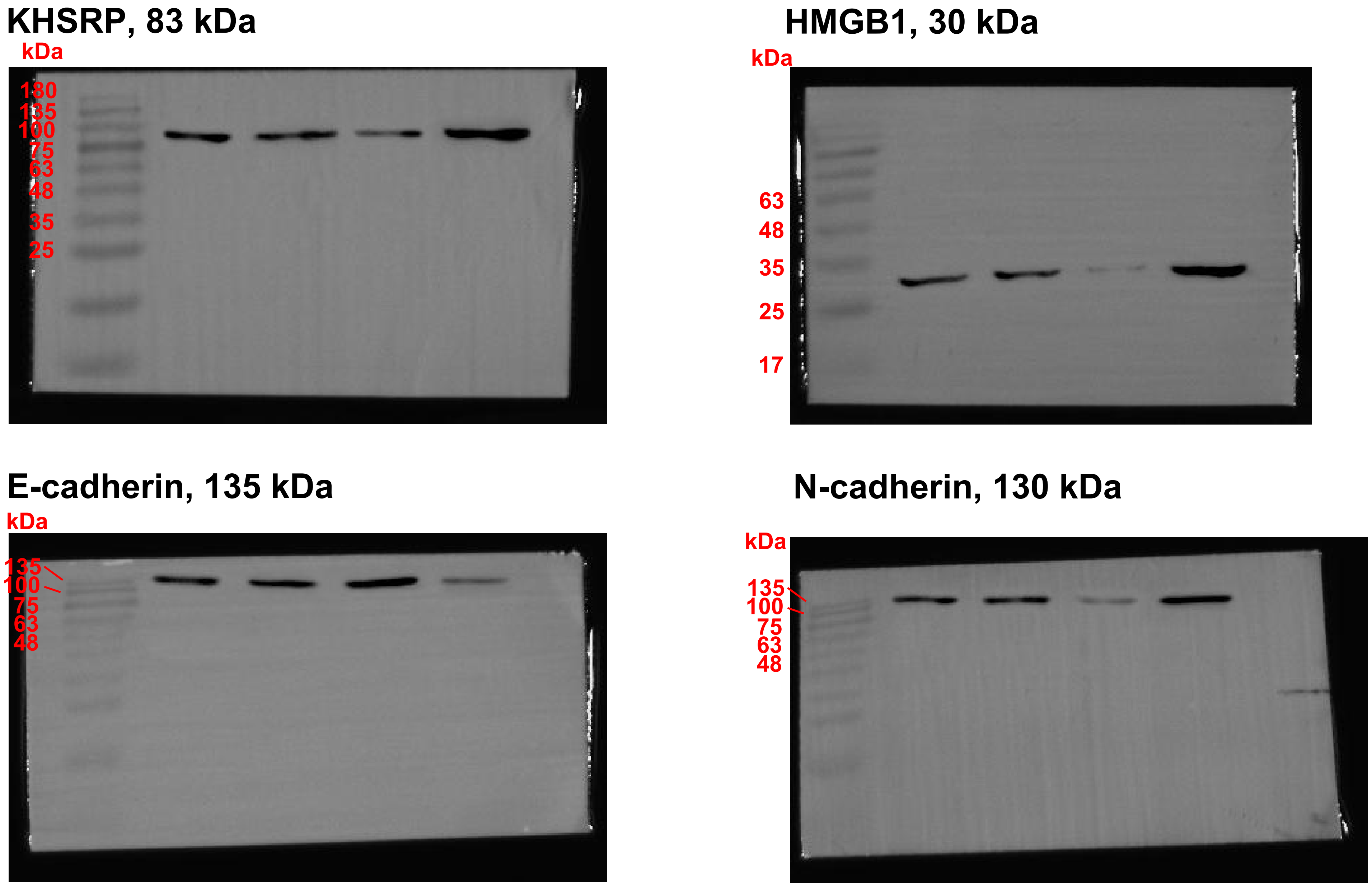
KHSRP, 83 kDa
kDa
180
135
100
75
63
48
35
25
HMGB1, 30 kDa
kDa
63
48
35
25
17
E-cadherin, 135 kDa
kDa
135
100
75
63
48
N-cadherin, 130 kDa
kDa
135
100
75
63
48

Figure 2B

Vimentin, 53 kDa

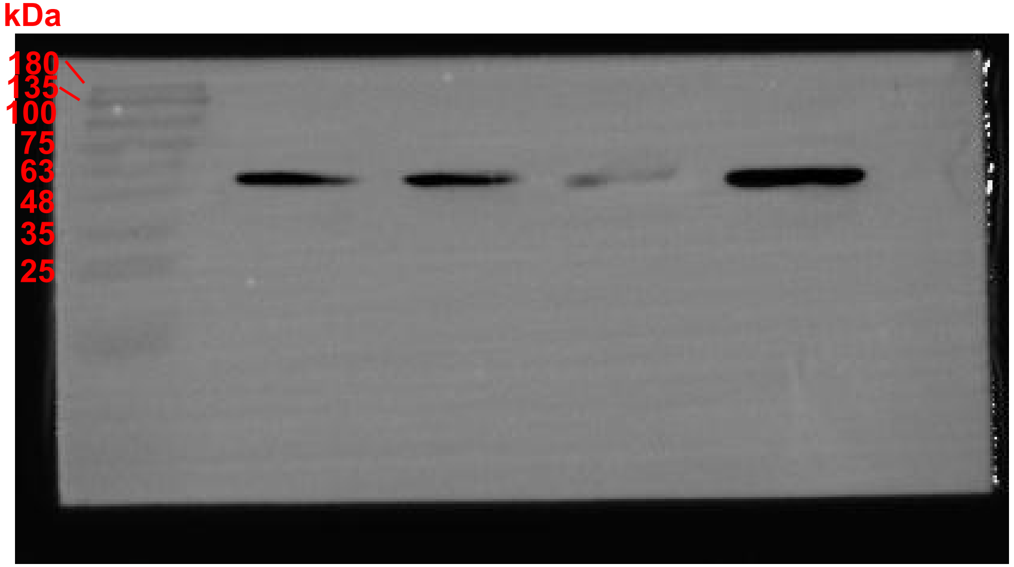

ki67, 395 kDa

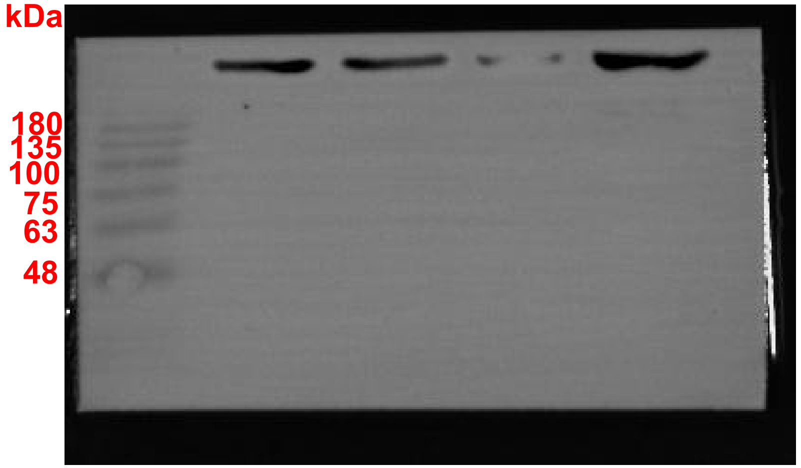

β-actin, 42 kDa

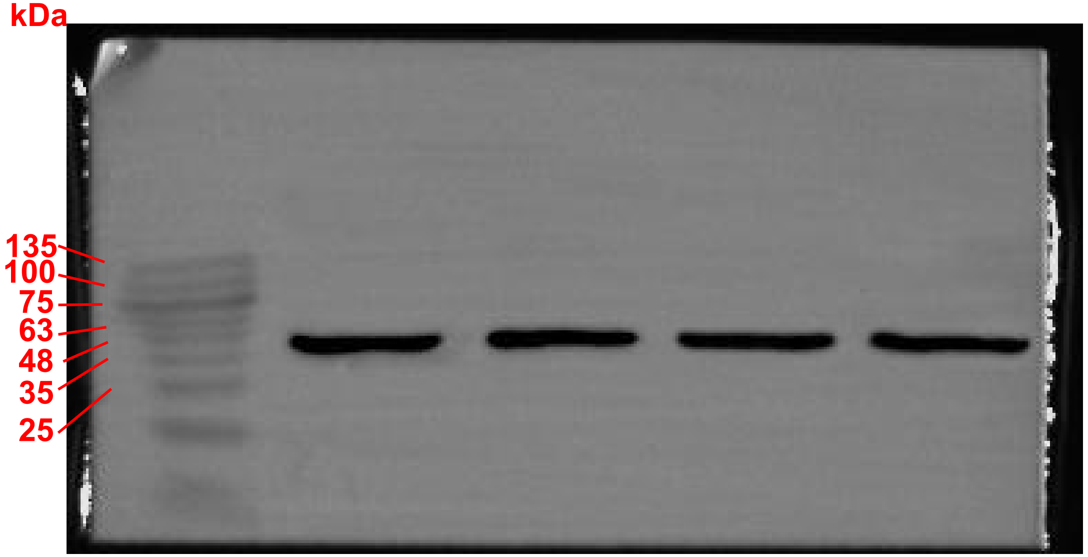

Figure 4C

KHSRP, 83 kDa

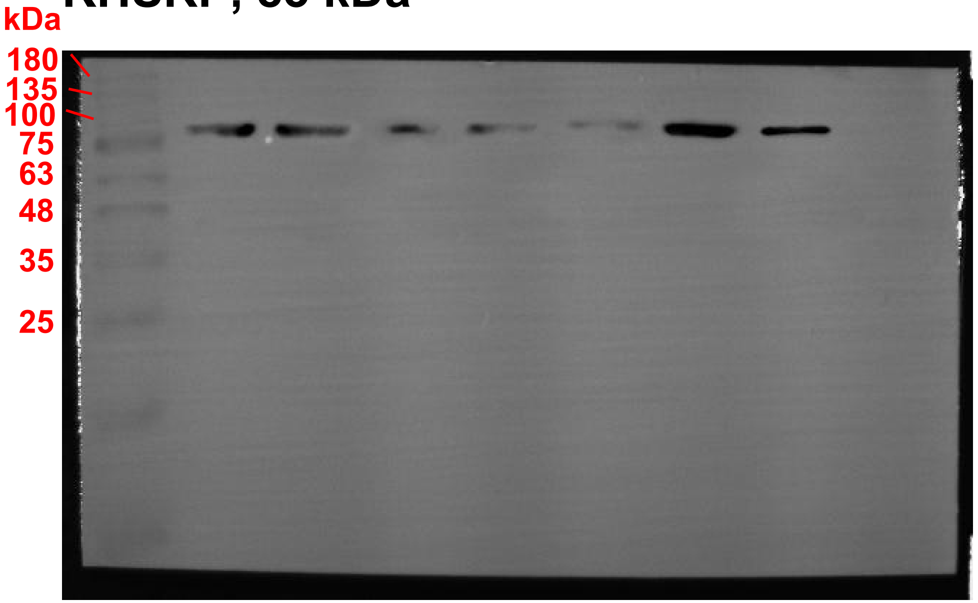

HMGB1, 30 kDa

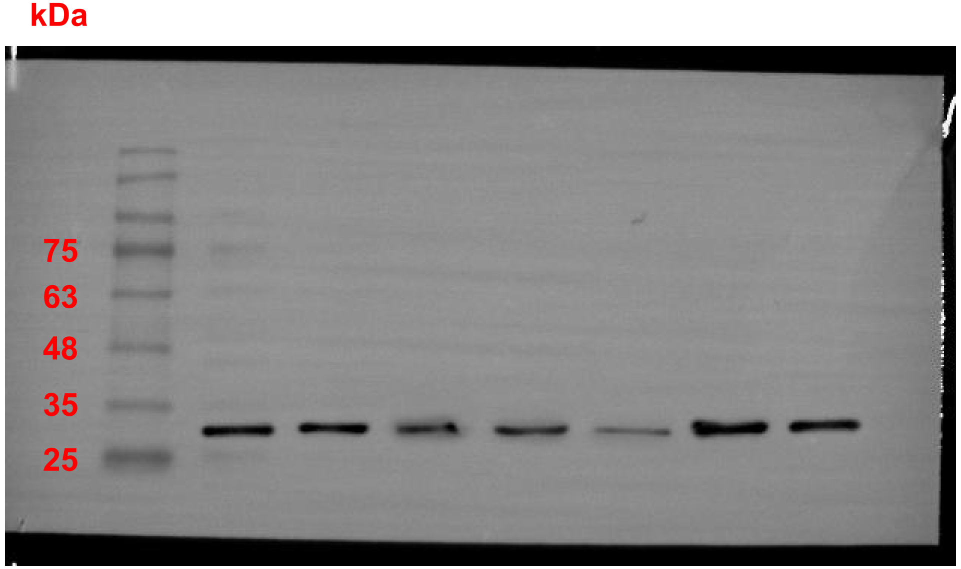

E-cadherin, 135 kDa

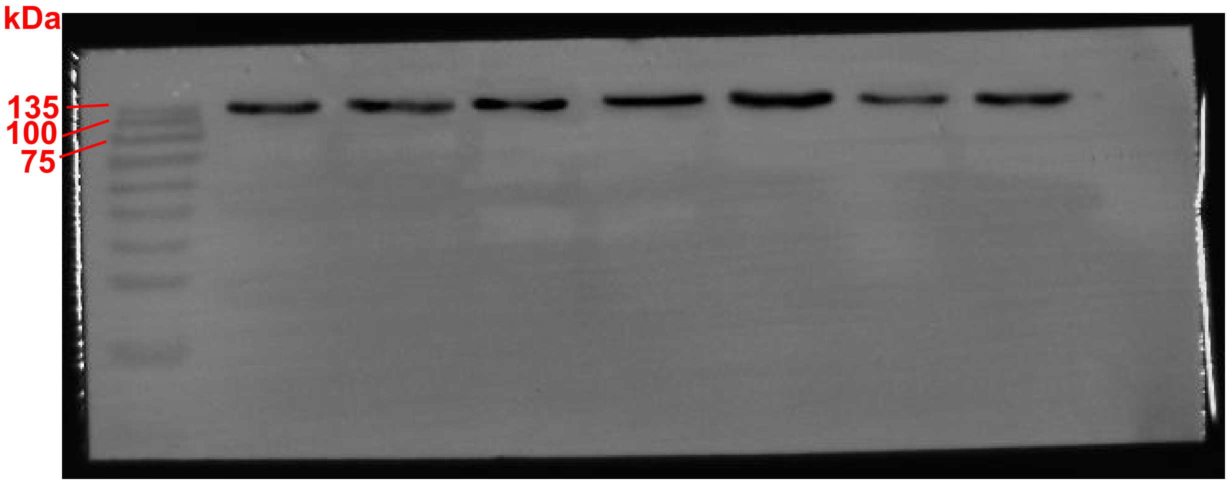

N-cadherin, 130 kDa

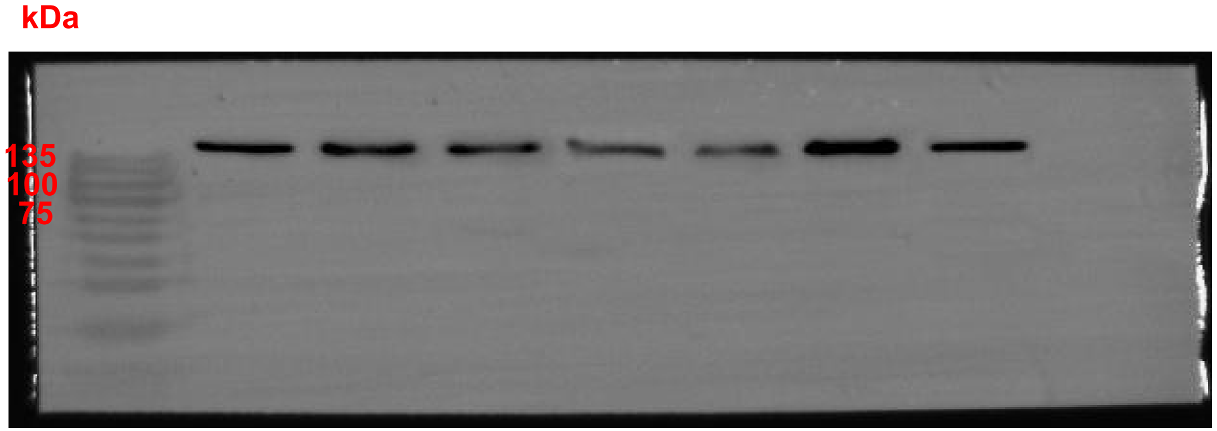

Figure 4C

Vimentin, 53 kDa

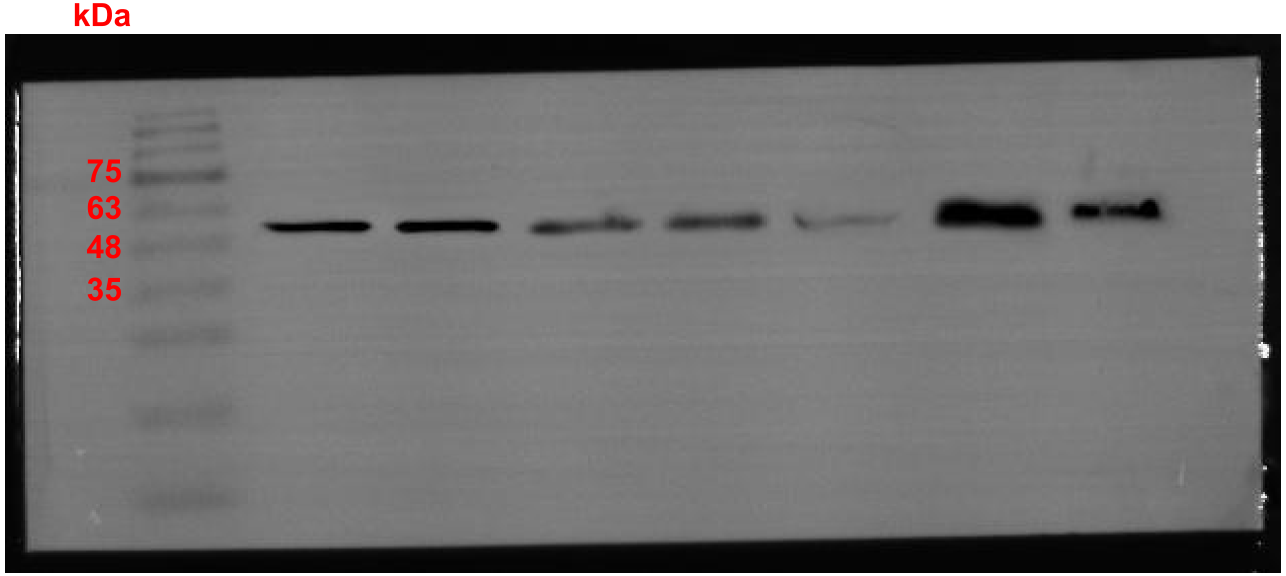

ki67, 395 kDa

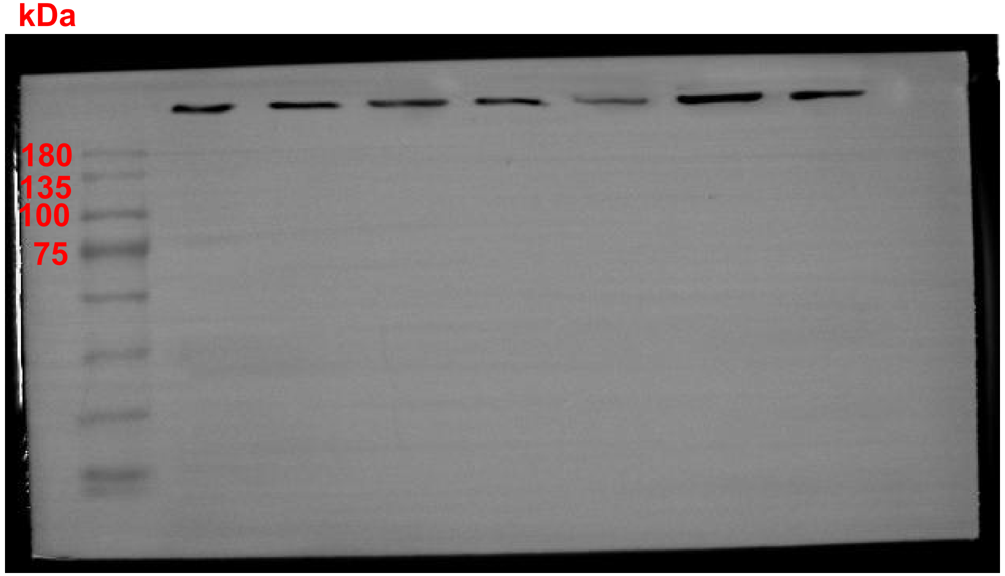

β-actin, 42 kDa

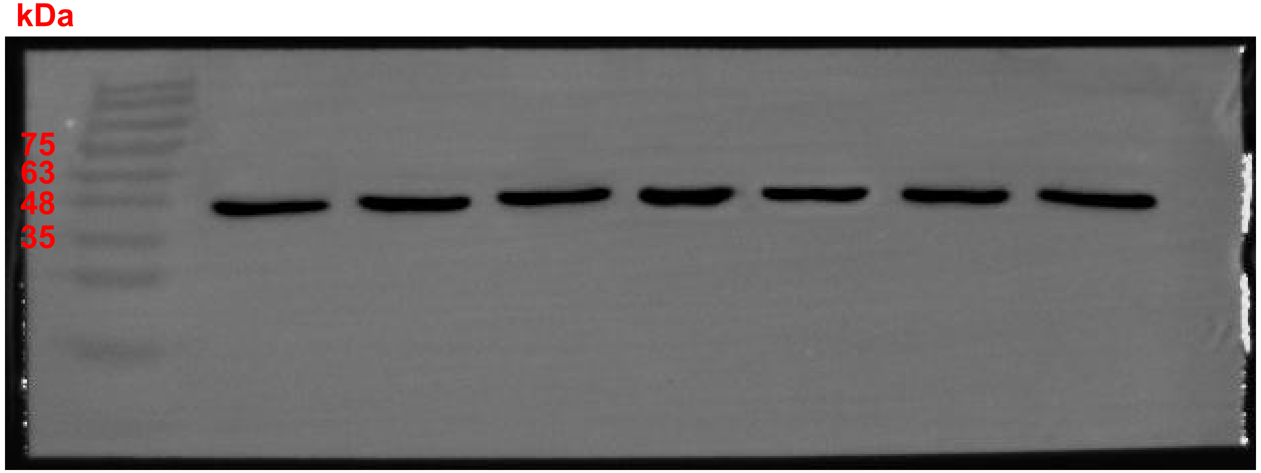

Figure 6A

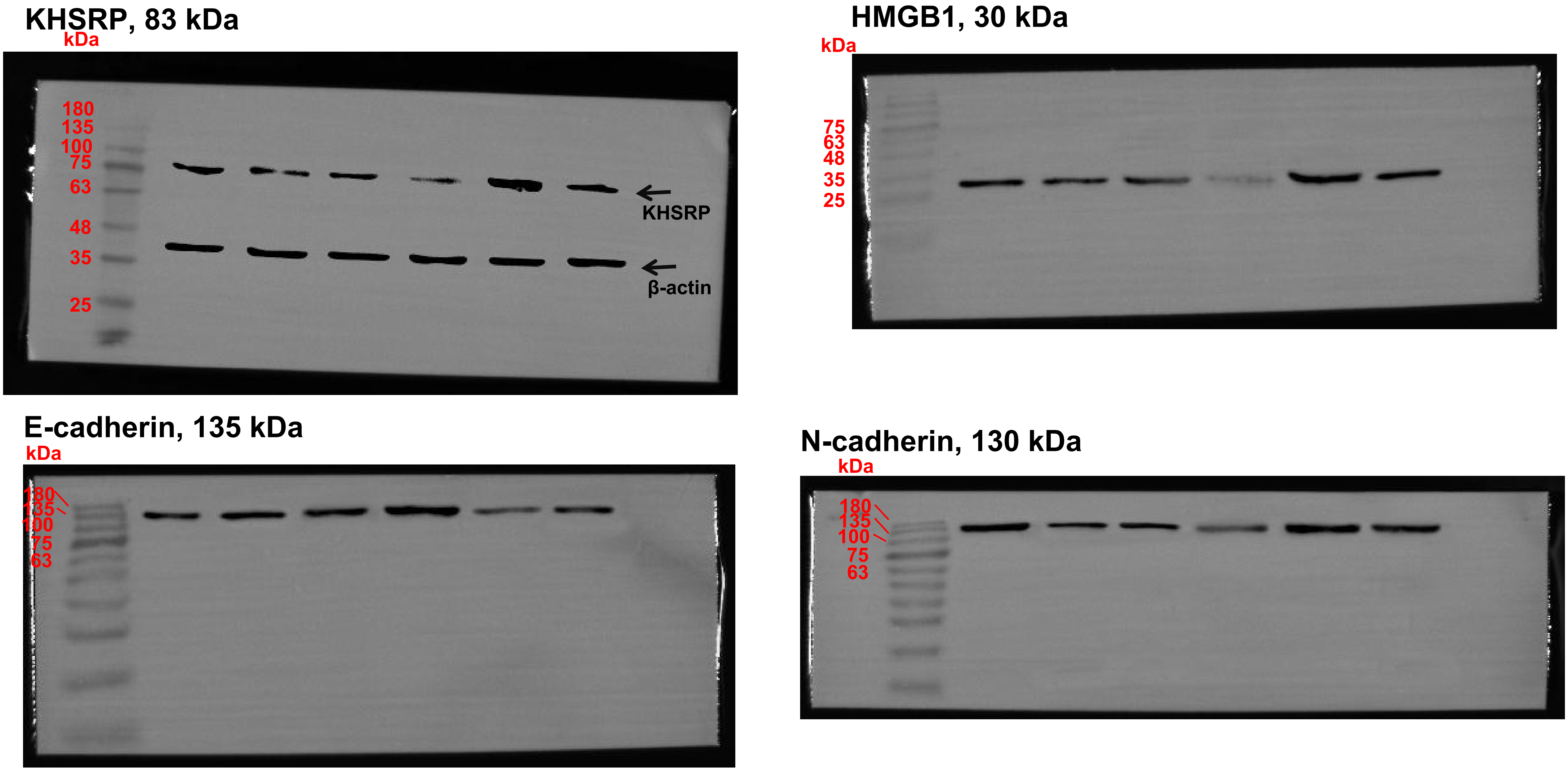

KHSRP, 83 kDa
kDa
180
135
100
75
63
48
35
25
KHSRP
β-actin
HMGB1, 30 kDa
kDa
75
63
48
35
25
E-cadherin, 135 kDa
kDa
180
135
100
75
63
N-cadherin, 130 kDa
kDa
180
135
100
75
63

Figure 6A

Vimentin, 53 kDa

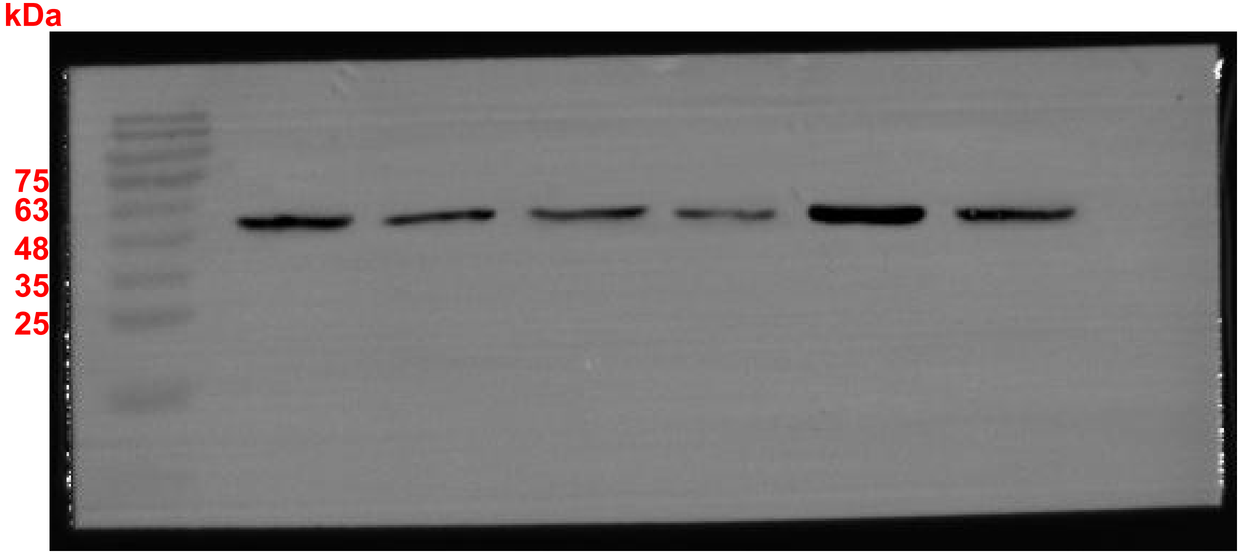

ki67, 395 kDa

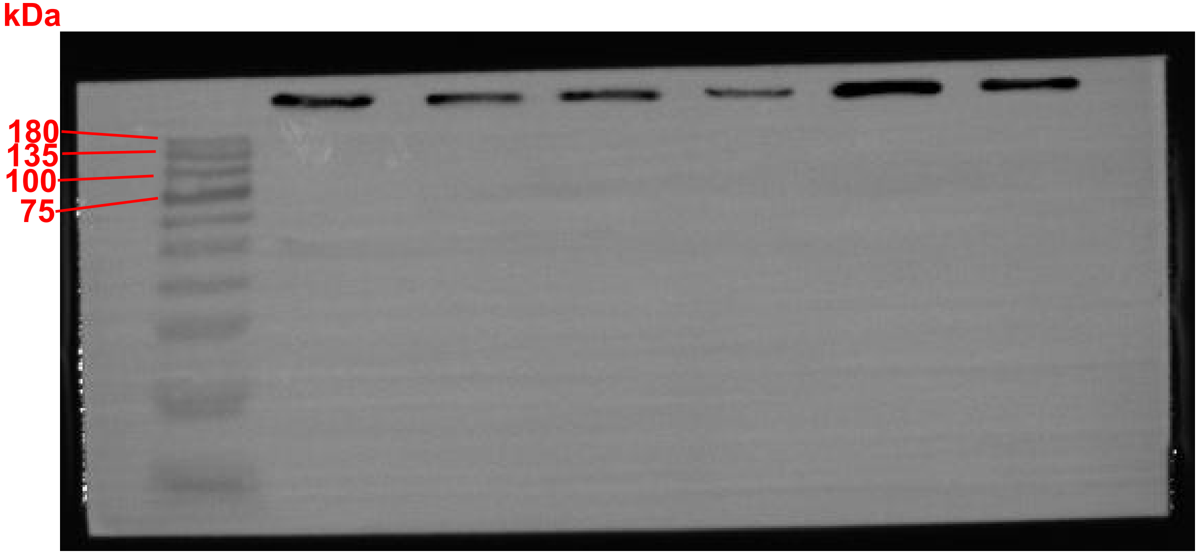

β-actin, 42 kDa

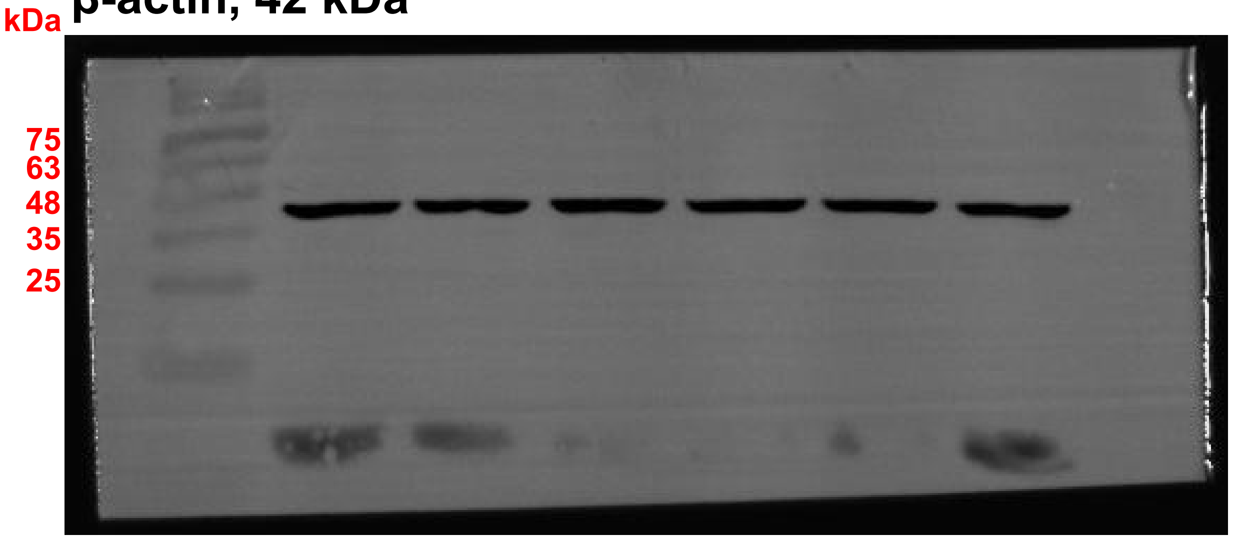

Supplement: Supplementary file 2 — Supplementary Material 2. [file 41065_2025_584_MOESM2_ESM.pdf]
